# Spasmodic Torticollis and Its Surgical Treatment1Read before the Section on Surgery, Buffalo Academy of Medicine, October 1, 1901.

**Published:** 1901-12

**Authors:** C. A. Hamann

**Affiliations:** Cleveland, O., Professor of Anatomy, Western Reserve University; Visiting Surgeon to Charity and City Hospitals


### BUFFALO MEDICAL JOURNAL.

VOL. XLI.—LVII. DECEMBER, 1901. No. 5.

### ORIGINAL COMMUNICATIONS.

### Spasmodic Torticollis and its Surgical Treatment.[1]

By C. A. HAMANN, M. D., Cleveland, O.,
Professor of Anatomy, Western Reserve University; Visiting Surgeon to Charity and City Hospitals.

WE designate as spasmodic torticollis an affection characterised by tonic or clonic spasms of certain muscles of the neck, by which the head and cervical spine are rotated in one or other direction, or drawn directly backward, depending upon the degree and extent of muscular involvement.

This disease is to be regarded as totally distinct from congenital wry-neck, from rheumatic torticollis, and from the torticollis due to cicatricial contraction, myositis or disease of the cervical vertebræ. It is an affection primarily of the nervous system, either central or peripheral, and in its manifestations is allied to facial spasm and to writer's cramp.

SYMPTOMS AND CLINICAL HISTORY.

The first evidences of its existence, as a rule, are spasmodic contractions of the sterno-cleido-mastoid muscle. Associated with these, or perhaps preceding them, there may be painful or uneasy sensations in the neck, occiput, post-auricular region, shoulder or arm. Cases have also been described in which there was at first dizziness, and others in which there was a tendency to deviate to one side (Gowers).

The spasms gradually become worse. They may be present only during excitement or when the patient is tired. In a very few cases the disease is fully developed in a very short time—say a few days or a week. The head is turned to one side or other, depending upon which sterno-cleido mastoid is involved. As is well known the action of this muscle is to rotate the head so that the chin looks to the opposite side and upward; the head is

1. Read before the Section on Surgery, Buffalo Academy of Medicine, October 1, 1901.

inclined to the same side. The contractions may be tonic or clonic. In the latter form the frequency of the contractions varies greatly. Irritation, as by compressing the muscle may bring on spasm; the muscle stands out prominently, and in time may become hypertrophied. The spasms cease during sleep.

In one of my cases the patient could with difficulty get no sleep, however, owing to her head boring itself into the pillow, as she expressed it. A common occurrence is to find a combination of the tonic and clonic spasms. Rarely both sterno-cleido-mastoids are involved. It may be mentioned in this connection that the usual statement that contraction of both sterno-cleido-mastoids at once causes flexion of the head is incorrect; it produces retraction owing to the fact that the muscles are inserted posterior to the point of articulation of the occipital bone with the atlas. This fact was long ago pointed out by Hyrtl. The German name "Kopfnicker" is therefore an inappropriate one for this muscle.

After a time other muscles participate in the contractions; this progressive involvement of certain other muscles is seen in most cases, and is one of the most disagreeable features of the disease. Probably by the time that the patient presents himself for treatment, the process will have passed beyond the sterno-mastoid.

In a large group of cases, the sterno-mastoid on one side and the splenius capitis and other rotator muscles of the other side are involved. The contraction of these posterior cervical muscles can be seen and felt, and one should never neglect to examine them in cases of spasmodic torticollis. Contractions of the sterno-mastoid of one side and the splenius of the other, have the same effect as regards rotating the head. Usually the head is drawn backward in such a case, owing to the greater strength of the posterior muscles. The other muscles which in time become involved, are the trapezius, complexus, trachelo-mastoid, obliqui, recti capitis postici, platysma and even the scaleni.

When the post-cervical muscles on both sides contract, the head is drawn backward—a condition known as retro-colic spasm. Occasionally the sterno-mastoid and posterior muscles of the same side are the seat of the spasm.

According to Gowers the muscles that turn the face to the left are much more often the seat of contraction than those which turn it to the right. It so happens that in all the cases of which I have notes—four in number—the face was turned to the left. The frequency of the contractions varies. However, it is rare to find them absent for more than a few minutes. Excitement increases their number and intensity. In many cases there is more or less tremor, so that there is a nodding movement of the head, or combined with the nodding movements, lateral motion. Other muscles than those of the neck may become involved, as those of the face or arm.

When there is much spasm, the patient is, of course, very uncomfortable; the muscles become fatigued, and there is apt to be neuralgic pain. As time goes on, the condition of the patient becomes almost unendurable, and some persons become tired of existence and are willing to undergo almost anything for the sake of relief. Work is impossible and pleasures cannot be participated in. Patients attempt to hold the head with the hands, or rest it against a chair.

The gradual progressive course of the disease may cease after a time and the patient's condition may remain unaltered. In a few cases the spasms cease spontaneously, or become much less intense. It may be said that the disease does not lead to any other serious disturbance of the nervous system, nor does it shorten life.

*Etiology.*—A number of causes of this disease are mentioned by writers—perhaps we had better say "preceding conditions," for the actual etiology is by no means always apparent. It is much more common in adults than in children.

Sex is a predisposing cause, for the affection is much more common in women than in men, according to Gowers. Osler makes the opposite statement. It is found that neurotic persons, or those with a family history of nervous affections or insanity sometimes become subjects of this disease. Melancholia, hysteria, depressing emotions, tremors and spasms of various sorts, worry and anxiety may precede it. In some cases there is a history of a chronic disease or loss of health and strength. Injuries precede it in a few cases.

A cause, which, in my opinion, is a very important and common one, is prolonged and excessive, or oft-times repeated contractions of the sterno-mastoid and other rotator muscles. Thus Annandale records a case of a girl, who "in the course of her work as a weaver, turned her head rapidly first to one side and then to the other, but especially to the left, and in the spasm the head was inclined and rotated to the left side"—Gowers. Walton lays stress on the influence that certain ocular defects, as oblique astigmatism and muscular insufficiencies have in producing spasmodic wry-neck. These defects produce a habit of turning the head in certain directions and spasmodic contractions of the muscles gradually come on.

In two of my cases, oft repeated contractions of one sterno-mastoid certainly seemed to be the causal factor. In one of these cases, the patient was deaf on the left side, and in the course of her occupation as a saleswoman, repeatedly turned her face to the left, so that she might hear with her right ear; gradually the involuntary, intermittent contractions of the right sterno-cleido-mastoid muscle came on.

In another case, the patient, who was an engineer on a switch engine, had to turn his head many times a day so as to look back over his left shoulder. In time spasmodic and involuntary contractions of the right sterno-mastoid developed.

The causal relations of repeated voluntary muscular contractions in these two cases seem to me quite clear. Whether an irritable state of the muscles alone is set up in such cases, or whether the protracted excessive use of the centers of rotation in the cerebral cortex leads to an exaggerated irritability of these centers is not positively known.

Gowers refers to a person in whom the spasms were brought on by the patient's nodding vigorously while speaking; this habit had existed for many years. Walton mentions the possibility that toxins, acting upon the cortical cells, may produce the disease, and he alludes to a case, recorded by Simon, in which malaria was the causative factor; the patient was cured by the administration of quinine.

Some cases of spasmodic torticollis are of hysterical origin—or at any rate are associated with other hysterical stigmata. Distant peripheral irritation, as by intestinal parasites, is said to have been the cause in a few cases.

*Pathology and Pathogenesis.*—This is a very obscure part of our subject. I know of no cases in which a careful post-mortem examination has been made, with the exception of one, referred to by Dana. I have only seen a very brief report of his case in a discussion in the American Neurological Association. Dana found a "chronic leptomeningitis of the convexity, with some degeneration of the cells." The fact that the disease is not fatal explains the non-existence of post-mortem findings. In reality therefore we can only surmise the pathology of the affection, though there are certain facts which point to the correctness of the view that it is a disease of cortical origin, in some cases at least. A lesion to produce the symptoms described, must be one of an irritative character, for a destructive lesion would, of course, cause paralysis. As Mills points out, such a focal lesion could also be in the upper portion of the cord, in the membranes, bones or nerves outside of the cord, but intraspinal, in the spinal accessory nerve, in the posterior part of the medulla, or in the nerves which communicate with the spinal accessory, as the second and third cervical. One can imagine that irritation of the upper cervical nerves, which are in communication with the spinal accessory, might lead to spasmodic contractions of the sterno-mastoid. Histologic examination of excised portions of the nerves has not revealed any abnormality.

The statement has been made above that long-continued and oft-repeated contractions of the rotator muscles of the head may lead to tonic or clonic contractions. It is known that such excessive use of muscles may lead to atrophy and degeneration, as is seen elsewhere in the body, in some forms of muscular dystrophy. In other cases, overuse leads to spasm, sometimes accompanied by pain, as witnessed in writer's cramp. In cases of atrophy as well as in spasm from overuse of muscles, the real seat of the trouble is in the nerve centers, and the most commonly held theory of the position of the lesion in spasmodic torticollis, is that it is in the cortex cerebri—in the centers for the movements of the head. The correctness of this theory, however, yet remains to be proven. Gowers alludes to the possibility that disturbance in the lower part of the pons may produce the disease, for it seems that certain structures here have an influence in the rotation of the head.

It is obvious that we cannot locate the lesion in the muscles themselves. When we make the statement that there is present somewhere an abnormal state of the nerve cells, which results in irregular and uncontrolled discharge of nerve-force, we do not really give an explanation of the etiology and pathogenesis of the disease, for we do not know the nature of the process nor can we locate it. Possibly the changes are such that microscopic examination cannot reveal them.

*Diagnosis*—The diagnosis of the affection under consideration is in the vast majority of cases simple. The congenital form of wry-neck, the rheumatic and other forms are easily differentiated. They are entirely different. There are certain forms of spasmodic rotation of the head around a vertical axis, which should be distinguished from accessorius spasm. I refer more particularly to the *tic rotatoire* of authors. In this affection, the head is turned intermittently from right to left and from left to right, there being no deviation of the chin or lowering of the mastoid process. Such movements may be produced by contractions of the small rotator muscles forming the suboccipital triangle. While these muscles do become involved in longstanding cases of spasmodic torticollis, still they are not at first the seat of contractions, and *tic rotatoire* is not the same affection as spasmodic wry-neck.

In the diagnosis of the disease under consideration, we should determine, if possible, just what muscles are involved in addition to the sterno-mastoid. A knowledge of the anatomy and physiology of the cervical muscles is, of course, essential in this determination. I have already alluded briefly to the symptoms produced by contractions of the several post-cervical muscles, and mentioned that the contractions can often be seen and felt. As stated above, the most frequent combination with sterno-mastoid spasm is that of spasm of the opposite splenius.

*Prognosis.*—The prognosis is in general unfavorable, as far as cure is concerned, *i.e.*, it is unfavorable in most cases unless the patient will submit to operative measures. Cases of hysterical origin may recover spontaneously.

The more severe the spasms, the longer the duration, the more muscles that are involved, the less the likelihood that much benefit can be obtained from treatment.

*Treatment.*—To describe the various forms of treatment that have been resorted to in this affection would be a wearisome and unprofitable task. Most drugs in the Pharmacopœia that have, or are supposed to have, an effect on the central or peripheral nervous system have been used. Some writers claim to have cured cases with certain drugs—others deny the efficiency of these same drugs. From what I gather from the literature, and from what I have seen myself, I have come to the conclusion that there are no drugs whatever, the administration of which results in permanent benefit. Electricity is useless. Mechanical support cannot be borne as a rule.

H. J. Hall reports two cases, one of which was cured, the other much benefited by gentle spring pressure on the back and sides of the neck, this was supplemented by gymnastic exercises designed to improve the general poise and to secure good muscular control throughout the body."

The rest-cure has been resorted to, and according to some with good results. Others find it of no avail. External applications, and the use of the cautery are not successful. There seems to be no question that massage has helped some patients. Of course, the removal of apparent causes should be the first part of the treatment.

It is well that the various medicinal, mechanical and hygienic measures be given a trial, however. In mild and early cases, they may be of avail. In severe and long-standing cases, the proper treatment and the one that offers good chances for cure, or at any rate improvement, is by operation, either on the nerves or the muscles, or on both. A sufficiently large number of cases is now on record to justify the above statement.

The operative treatment of spasmodic torticollis was originated by Campbell de Morgan, who in 1866 cured a case of this disease by excision of a portion of the spinal accessory nerve. Keen, in 1891 devised a method of operating on the posterior cervical nerves, as did also Noble Smith, in the same year. Gardner claims priority over Keen in the suggestion of the method. In 1895 Kocher, through his assistant, De Quervain, made known his method of operation on the muscles. This, in brief, is a history of the surgical treatment of spasmodic torticollis.

It may be stated here that nerve stretching and simple neurotomy have been shown to be useless, and are therefore discarded. Tenotomy of the sterno-cleido-mastoid, it is needless to say is of no avail.

Resection of a portion of the spinal accessory nerve is a comparatively simple operation. The incision is made along the anterior border of the muscle, extending from a point about half an inch below the tip of the mastoid process, downward for an inch and a half. After exposing the border of the muscle and carefully checking the hemorrhage, the nerve is sought for in the loose tissue exposed. It passes under the border of the muscle, from an inch to an inch and a half below the tip of the mastoid. A landmark of value is the transverse process of the atlas, which the nerve crosses. The posterior belly of the digastric is also seen; the nerve comes from beneath it. It is well to bear in mind the proximity of the internal jugular vein; sometimes the nerve passes over it, sometimes under. When the spinal accessory is not at once seen, its position can be determined by irritating the tissue with a blunt instrument, as Richardson suggests. The instant that the instrument strikes the nerve the sterno-mastoid contracts. An inch or more of the nerve should be excised. In one case in which I attempted to find the nerve on the cadaver, I was unsuccessful, and the dissection revealed the fact that there was no spinal accessory, or rather that its place was taken by a branch from the second and third cervical nerves. Dandridge records a case in which he failed to find the nerve. In one of my cases I found it entering the muscle three-fourths of an inch lower than normal.

Though the sterno-mastoid receives filaments from the second and third cervical nerves, excision of the spinal accessory completely paralyzes it, the muscle becomes relaxed and subsequently no longer contracts. It undergoes atrophy in time. By this procedure, cases in which the sterno-mastoid is the only muscle which is involved, can be cured. No perceptible interference with the movements of the head results, and the paralysis of the upper part of the trapezius gives no inconvenience. Possibly the shoulder drops slightly. A minor point in the operation that I failed to mention above, is the avoidance of cutting the auricularis magnus nerve, which lies in the superficial fascia, obliquely to the line of the incision. It is well to refrain from cutting it, for the patient may complain, as did one of mine, of a disagreeable numbness of the external ear. When, in addition to the sterno-mastoid, the splenius and other muscles of the other side of the neck are involved, it will be necessary to excise their nerves also, in order to secure satisfactory results.

Keen's method affords a good way of reaching these nerves. I will attempt a description of this procedure, based on numerous operations on the cadaver, and two on the living subject.

The patient is laid on his abdomen, with the head hanging over the end of the table. This places the anesthetiser in an uncomfortable position. A transverse incision, three inches long across the neck, about on a level with the lobule of the ear, divides the skin, superficial and deep fasciæ, and exposes the trapezius. This muscle is then cut across, and upon raising the upper portion of the muscle, the occipitalis major nerve is looked for. It will be found near the median line, piercing the complexus and entering the trapezius. It is a good-sized nerve and can be easily found, as a rule, It is to be freed from its surroundings, down to where it pierces the complexus. The complexus is then cut transversely. Care is to be taken that the nerve is not divided. The upper portion of the complexus is then raised and the suboccipital triangle exposed. In this triangle near the outer angle (the one formed by the obliquus inferior and superior) the branches of the suboccipital nerve are sought for. They are small, and, in my experience the most difficult of the three upper cervical nerves to find. There is more or less areolar tissue in the suboccipital triangle; this, and the bleeding from the rather numerous small veins here found, render it difficult to find the nerve. When it is found the nerve is to be traced down close to the spine and cut; the fact that the vertebral artery is also in this triangle should be remembered.

The operator returns to the occipitalis major nerve, follows it around the lower border of the obliquus inferior, and after tracing it beyond the point where the external or muscular branch is given off, divides the posterior primary division of the second cervical nerve. The lower portion of the complexus is then raised and the internal branch of the posterior division of the third cervical nerve is looked for. It will be found under the complexus, about three quarters of an inch below the second cervical. The internal branch of the posterior division of the third nerve is then followed toward the spine, to beyond the point where the external or muscular branch is given off and the whole posterior division is then cut close to the spine.

By these means, the nerve supply to the muscles concerned in the production of the deformity is cut off. The wound is then sutured. The divided muscles are not united by suture. No drainage is necessary if hemostasis has been thoroughly effected. Noble Smith made a vertical incision, from the occiput downward for three inches; one inch to the outer side of the spinous processes.

Experience has shown that such an operation even if bilateral, does not lead to interference with the movements of the head, nor does it cause an inability to hold the head erect.

Kocher's operation consists in a transverse division of the muscles involved. He insists upon the importance of subsequent treatment by manipulations and gymnastics. Section of the muscle does not produce paralysis, rigidity, or atrophy. Of twelve cases operated upon by Kocher, seven were completely cured, and at the time of the report five were still being treated.

De Quervain's theory is that section of the muscles acts as a suggestion to the cerebral cortical centers for rotation, that is to say, the spasms having been inhibited for a time by the operation, they do not return subsequently--the habit having been broken.

Richardson in a number of cases has combined muscle section with neurectomy, with good results.

My own operative experience is limited to two cases, which will next be related.

Miss K., aged 52; a saleswoman by occupation noticed an intermittent turning of the head to the left in August, 1895. She had for many years been deaf on the left side, and was in the habit of turning the head to the left in order to hear better, using therefore the right sterno-mastoid to excess. The movements became quite annoying. For the next few months I resorted to various drugs and to electricity, without benefit. In September, 1896, I excised a portion of the right spinal accessory. Of course, the muscle now no longer contracted and she was improved. However her head still turned to the left and was drawn backward, and it was then found that the left splenius and complexus were the seat of spasm.

For a year or more she went without treatment. She was then placed in the hands of a distinguished neurologist, who tried the rest-cure, drugs, cauterisation, and a collar. No benefit resulted, indeed the rest-cure weakened her. She became worse and worse. All that she could do was to sit in a chair, and brace her head against the back. She became an invalid, shunned society and could participate in no pleasures.

In the summer of 1900 it was found that the posterior cervical muscles upon both sides were the seat of the spasm. This spasm was for the most part tonic, with exacerbations at intervals, in which the spasms were clonic. The head was drawn almost directly backward, the so-called retro-colic spasm. She was in such a miserable state, that she was willing to undergo anything for the sake of relief.

I decided to excise the posterior division of the three upper cervical nerves and also to cut the muscles. At the operation, I was unable to find one of the sub-occipital nerves, but found all the other nerves. I also cut the trapezius, splenius capitis, complexus, trachelo-mastoid, and obliquus inferior on each side.

The wound healed by first intention. When she recovered from the operation there was no more spasm. The head was held slightly backward, and more or less tremor was present. She could move her head quite well in all directions, and expressed herself as much improved. At the present time, fifteen months after the operation, there exists some tremor. There is, of course, no retrocolic spasm. The patient is able to attend to her work in the house, to do needle-work, to go driving and to travel.

A second case was Miss L., aged 32 years, referred to me by Dr. H. S. Upson.

The patient was a spare neurotic woman. She had been very "nervous" as she expressed it for two years or more. Worry and grief seemed to be the only cause that could be found for the torticollis, which had existed for five months. She had been treated medically most of the time without improvement. There was, when I first saw her clonic contraction of the right sterno-mastoid, and of the left post-cervical muscles—the usual form, as I have mentioned above. The spasms were quite infrequent when she was lying down, but were severe when she sat up. Her right spinal accessory was excised, as were also the posterior primary divisions of the three upper cervical nerves on the left side. The muscles were cut. The result is that no more spasm exists, and her head does not turn at all.

In conclusion, I should like to submit the following:

1. The etiologic importance of frequently repeated muscular contractions in the production of the disease.

2. The uselessness of non-surgical measures—at any rate in advanced cases.

3. The safety of the operation, the absence of impairment of motion after them, and the good chance there is of obtaining a cure, or at least improvement.

4. I would urge the performance of radical and extensive operations—excision of the nerves and divisions of the muscles.

